# Supplementary material for: CRISPR/Cas9 Knock-Out in Primary Neonatal and Adult Cardiomyocytes Reveals Distinct cAMP Dynamics Regulation by Various PDE2A and PDE3A Isoforms
Source: Cells. 2023 Jun 4;12(11):1543. doi: 10.3390/cells12111543 (PMC10253201; doi:10.3390/cells12111543)
Supplement: Supplementary file 1 [file cells-12-01543-s001.zip › Supplementary figures and tables.pdf]

# Supplementary Figure S1

**A**

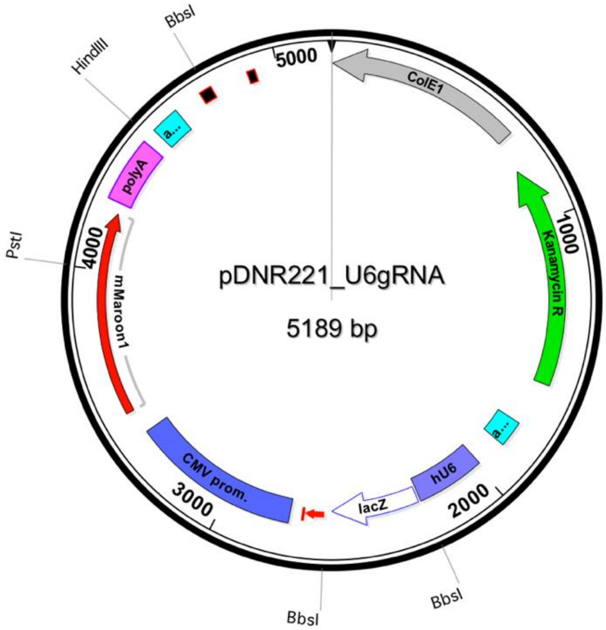


**B**

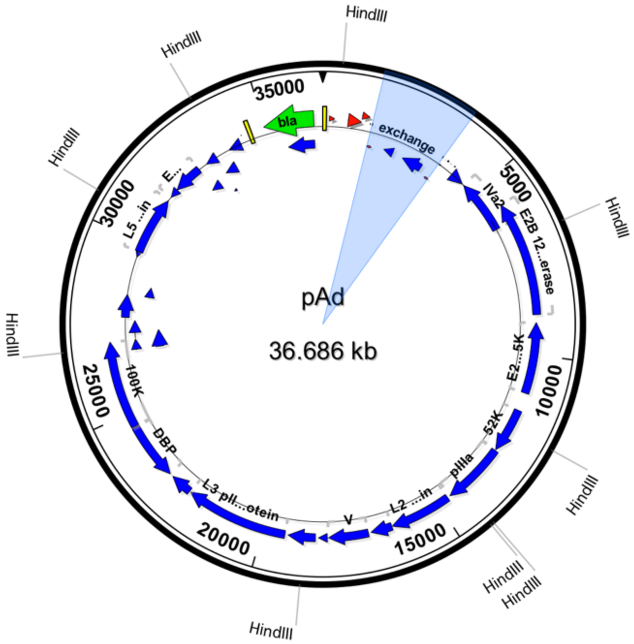


**Supplementary Figure S1. Schematic structure of the shuttle (pDNR221) and adenoviral (pAd) vectors used for gRNA adenovirus generation.** (**A**) Genetic cassette encoding LacZ under the control of the U6 promoter followed by cytomegalovirus (CMV) promoter and mMaroon1 sequences was cloned into pDNR221 vector between the attL sites (turquoise). Next, LacZ was exchanged by individual gRNA sequences via BbsI restriction site and corrected sequence/orientation was verified by sequencing. Full sequence of the construct can be found in supplementary files. (**B**) gRNA containing cassette from the pDNR221 vector was recombined into adenoviral vector using the LR reaction of the gateway cloning protocol as per manufacturer instructions.

# Supplementary Figure S2

**A**

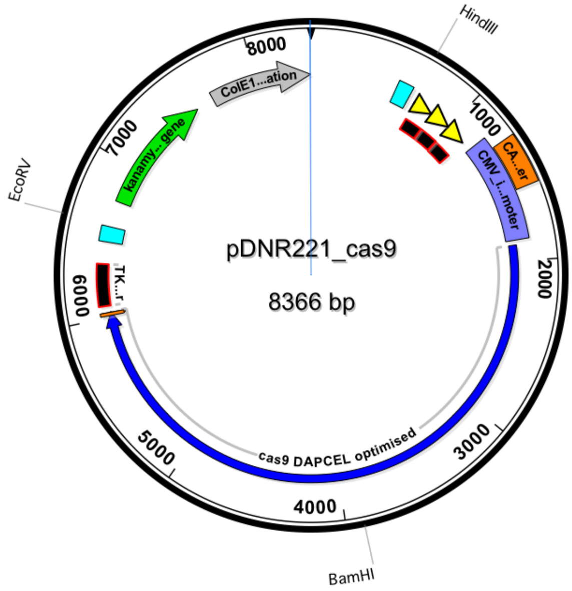


**B**

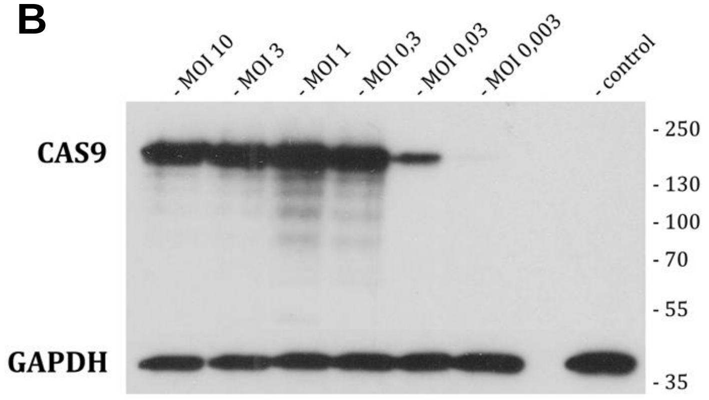


**Supplementary Figure S2. Cas9 expression in neonatal cardiomyocytes transduced with different amounts of Cas9 adenovirus. (A)** Schematic structure of the shuttle vector encoding Cas9. An optimized Cas9 sequence under the control of CMV promoter was cloned after three transcription stop signals (yellow arrows). Full sequence of the construct can be found in supplementary files. **(B)** Rat neonatal cardiomyocytes were transduced for 5 days with Cas9 adenovirus at different multiplicity of infection (MOI) and collected for immunoblot analysis. Representative blots, n=4.

# Supplementary Figure S3

**A**

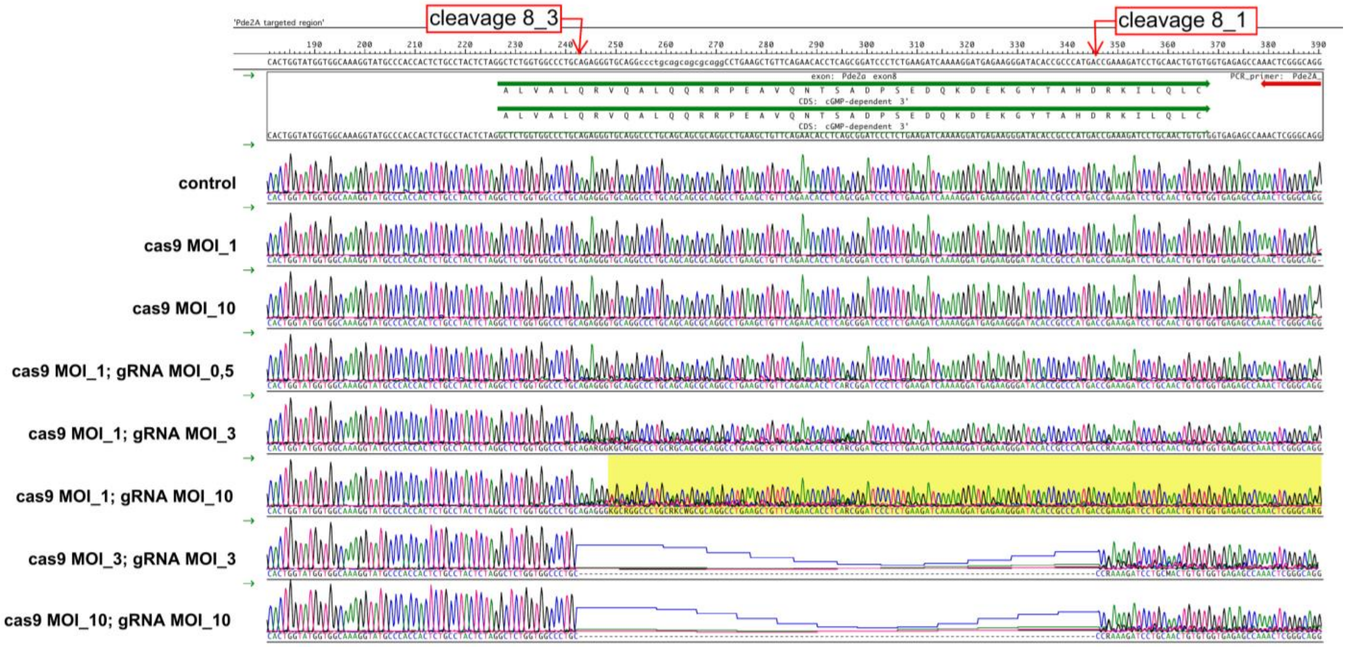


**B**

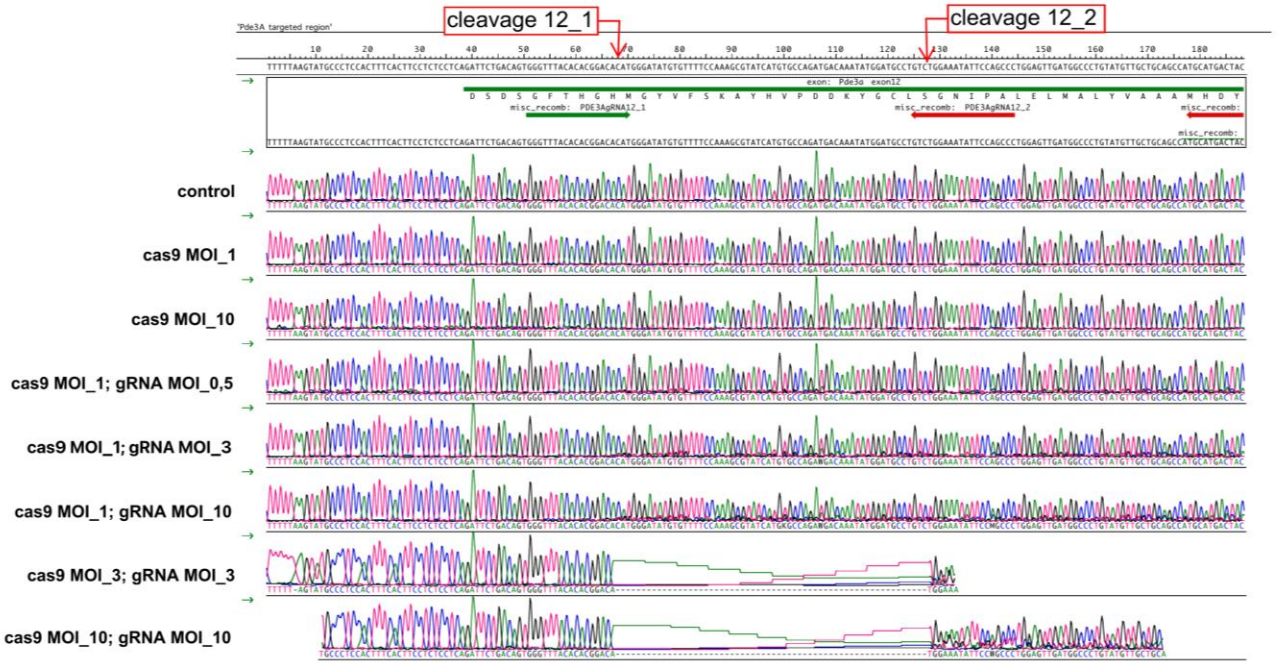


**Supplementary Figure S3. Genomic rearrangements in neonatal cardiomyocytes with PDE2A and PDE3A-KO.** Rat neonatal cardiomyocytes were transduced for 14 days with Cas9 plus gRNA adenoviruses for PDE2A-KO (**A**) or PDE3A-KO (**B**) as described in Figure 2. Targeted genomic loci of both PDEs were amplified by PCR and sequenced. The cleavage sites for CRISPR/Cas9 complexes with gRNA's for PDE2A and PDE3A are highlighted by arrows above. The MOI of adenoviruses for Cas9 and gRNAs are presented on the left. The 100% deletion of the targeted regions in PDE2A and PDE3A cardiomyocytes can be achieved at MOI 3 or higher.

# Supplementary Figure S4

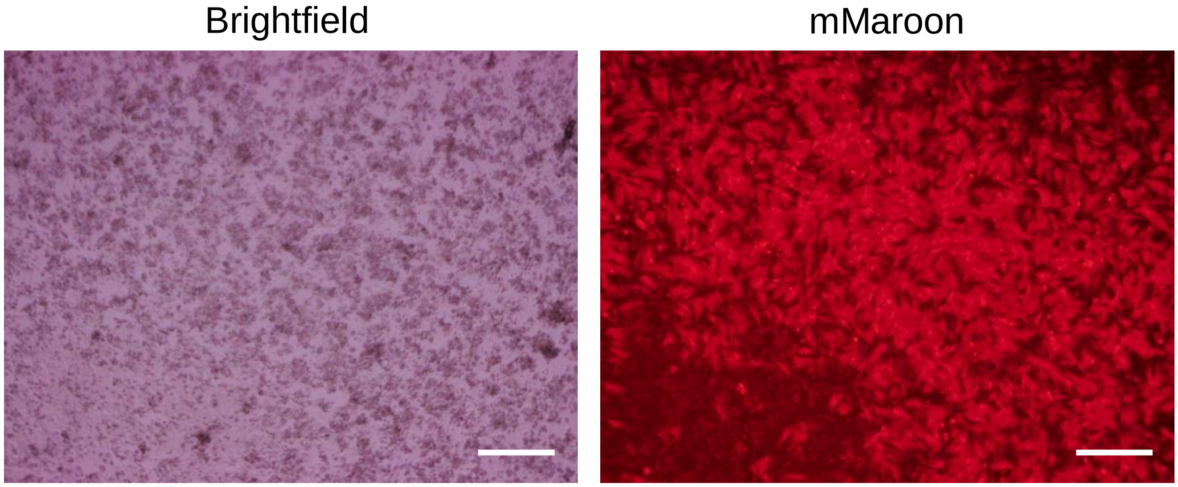


**Supplementary Figure S4. Control of expression for gRNA constructs in neonatal cells.** Neonatal cardiomyocytes were transduced for 3 days with Cas9 and PDE2A gRNA adenoviruses as described in Figure 3. mMaroon fluorescence was detected in red fluorescent channel of the Keyence BZ microscope. Representative images, n=10. Scale bar, 100 µm.

# Supplementary Figure S5

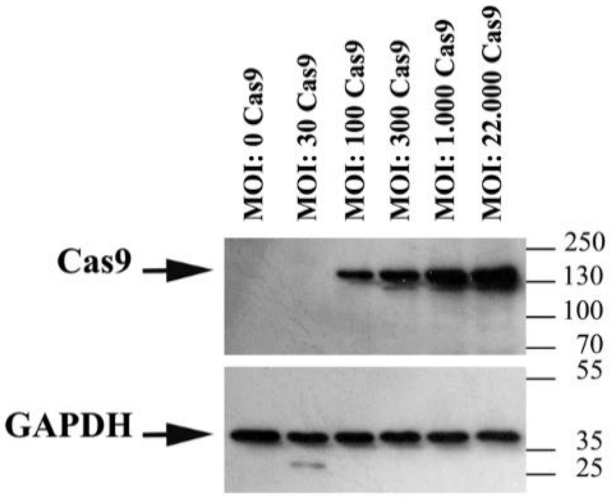


**Supplementary Figure S5. Cas9 expression in adult cardiomyocytes transduced with different amounts of Cas9 adenovirus.** Adult rat ventricular cardiomyocytes were transduced for 3 days with Cas9 adenovirus at different multiplicity of infection (MOI) and collected for immunoblot analysis. Representative blots, n=4.

# Supplementary Figure S6

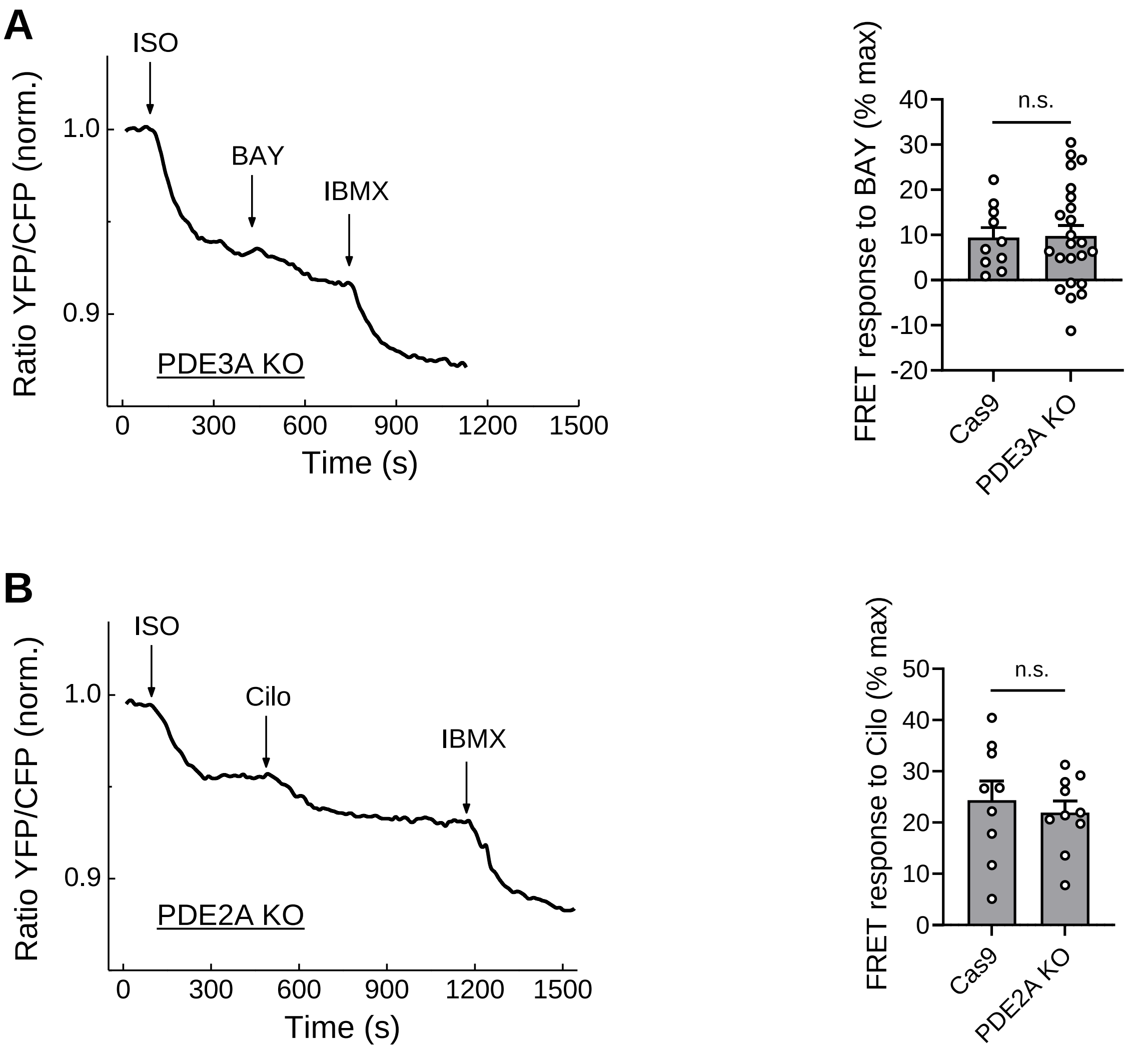


**Supplementary Figure S6. Control experiments performed to assess the effects of PDE2A knockout on PDE3A and *vice versa*.** Adult rat ventricular cardiomyocytes were transduced for 6 days with Cas9 or Cas9 plus gRNA adenoviruses as described in Figure 5. The effect of the PDE2 inhibitor BAY 60-7550 (100 nM) in PDE3A-KO cells (A) and of the PDE3 inhibitor Cilostamide (Cilo) in PDE2A-KO cells (B) were measured as described in Figure 5. Representative FRET traces and data analysis, n.s. – not significant by mixed ANOVA followed by Chi-squared test.

# Table S1

**Primer sequences used for RT-PCR of individual PDE2A and PDE3A isoforms.**

| Primer name | Primer sequence |
|---|---|
| PDE2A_totD1 | CCTGCAGGTGGTGGAGAA |
| PDE2A_totR2 | AGAGGGATCCGCTGAGGT |
| PDE2A_A1_D1 | GCCAGCAGGTCTTCCTCA |
| PDE2A_A1_D2 | GACCCCCTTGCACAGAAG |
| PDE2A_A1_R1 | AGGCTCAGCAAAGCATCC |
| PDE2A_A2_D1 | AGCATGGTCCTGGTGTTGC |
| PDE2A_A2_D2 | TACGAGCATGGTCCTGGTGT |
| PDE2A_A2_R1 | TTGGACAACAGCGATGAGG |
| PDE2A_A2_R2 | GAATTGGACAACAGCGATGA |
| PDE2A_A3_D1 | GGCAGATGATGAGGAGTGATG |
| PDE2A_A3_D2 | CAGGAGCCAGCAGTACCC |
| PDE2A_A3_R1 | GGCTTGAGGAAGACCTGCT |
| PDE2A_A3_R2 | AGGCTCAGCAAAGCATCC |
| PDE3A_totD2 | GGAGTTGATGGCCCTGTATG |
| PDE3A_totR2 | GGAACGGTCATTGTACAGCA |
| PDE3A_ A1_D1 | GATTCCGGGGTGGAAGAG |
| PDE3A_ A1_D2 | AGACCTTACCTGGCATACTTGG |
| PDE3A_ A1_R1 | CTCTCTTGTGGTCCCATTCTG |
| PDE3A_ A1_R2 | CGCCTCCTCCTCTTCCAC |
| PDE3A_ A23_D1 | GAAAGGAGAGCAAGATACCAATTT |
| PDE3A_ A23_D2 | GAAGGCAAGAAGCAAAGGAA |
| PDE3A_ A23_R1 | TCTCTTGTGGTCCCATTCTGA |

# Table S2

**Primer combinations used for RT-PCR of individual PDE2A and PDE3A isoforms.**

| Isoforms | Primer combinations | PCR product size |
|---|---|---|
| PDE2A all isoforms | PDE2A_totD1 + PDE2A_totR2 | 103 bp |
| PDE2A1 | PDE2A_A1_D1 + PDE2A_A1_R1 | 141 bp |
| PDE2A1 | PDE2A_A1_D2 + PDE2A_A1_R1 | 71 bp |
| PDE2A2 | PDE2A_A2_D1 + PDE2A_A2_R1 | 45 bp |
| PDE2A2 | PDE2A_A2_D1 + PDE2A_A2_R2 | 48 bp |
| PDE2A2 | PDE2A_A2_D2 + PDE2A_A2_R1 | 49 bp |
| PDE2A2 | PDE2A_A2_D2 + PDE2A_A2_R2 | 52 bp |
| PDE2A3 | PDE2A_A3_D1 + PDE2A_A3_R1 | 116 bp |
| PDE2A3 | PDE2A_A3_D1 + PDE2A_A3_R2 | 173 bp |
| PDE2A3 | PDE2A_A3_D2 + PDE2A_A3_R1 | 66 bp |
| PDE2A3 | PDE2A_A3_D2 + PDE2A_A3_R2 | 123 bp |
| | | |
| PDE3A all isoforms | PDE3A_totD2 + PDE3A_totR2 | 118 bp |
| PDE3A1 | PDE3A_ A1_D1 + PDE3A_ A1_R1 | 149 bp |
| PDE3A1 | PDE3A_ A1_D1 + PDE3A_ A1_R2 | 27 bp |
| PDE3A1 | PDE3A_ A1_D2 + PDE3A_ A1_R1 | 289 bp |
| PDE3A1 | PDE3A_ A1_D2 + PDE3A_ A1_R2 | 167 bp |
| PDE3A2 + PDE3A3 | PDE3A_ A23_D1 + PDE3A_ A23_R1 | 78 bp |
| PDE3A2 + PDE3A3 | PDE3A_ A23_D2 + PDE3A_ A23_R1 | 95 bp |
