# Supplementary material for: CRISPR/Cas9 Knock-Out in Primary Neonatal and Adult Cardiomyocytes Reveals Distinct cAMP Dynamics Regulation by Various PDE2A and PDE3A Isoforms
Source: Cells. 2023 Jun 4;12(11):1543. doi: 10.3390/cells12111543 (PMC10253201; doi:10.3390/cells12111543)
Supplement: Supplementary file 1 [file cells-12-01543-s001.zip › Supplementary File S1 pDNR221_U6gRNA.pdf]

```
LOCUS       pDNR221_U6gRNA          5189 bp    DNA     linear       06-APR-2023
FEATURES             Location/Qualifiers

     rep_origin      4..677
                     /note="ColE1 (pUC-type) origin of replication"
     gene            798..1607
                     /note="kanamycin resistance gene"
     misc_feature    1777..1876
                     /note="attL2"
     promoter        1986..2249
                     /label=hU6_promoter
     misc_feature    2252..2257
                     /label=BbsI
     gene            2416..2595
                     /gene="lacZ"
     CDS             2259..2595
                     /gene="lacZ"
                     /note="beta-d-galactosidase"
     misc_feature    2617..2622
                     /label=BbsI(1)
     misc_structure  2625..2701
                     /note="gRNA"
     terminator      2702..2707
                     /note=""
     enhancer        2755..3134
                     /label=CMV enhancer
                     /note="human cytomegalovirus immediate early enhancer"
     promoter        3135..3382
                     /label=CMV promoter
                     /note="human cytomegalovirus (CMV) immediate early
                     promoter"
     CDS             3475..4209
                     /dnas_title="mMaroon1"
                     /codon_start=1
                     /product="bright, rapidly maturing, monomeric far-red
                     fluorescent protein (Bajar et al., 2016)"
                     /label=mMaroon1
                     /translation="MVSKGEELIKENMHTKLYLTGTVNNHYFECTAEGEGKPYEGTQT
                     NRIKVVRGGPLPFAFDILAPCFMYGSKTFINHPPDIPDYFKQSFPEGFTWERTTVYED
                     GGTLTATQDTSLQDGCLIYNVQVRGENFPSNGPVMQKKTLGWEASTETLYPADGSLEG
                     RLDWALKLVGGGHLHCRLETTYRSKKPAKNLKMPGVYFIDRRLERIKEADNETYVEQH
                     EVAVARYCDLPSKLGHKLNGMDELYK"
     polyA_signal    4248..4472
                     /label=bGH poly(A) signal
                     /note="bovine growth hormone polyadenylation signal"
     misc_feature    4522..4621
                     /note="aatL1"

ORIGIN
        1 AACATGTGAG CAAAAGGCCA GCAAAAGGCC AGGAACCGTA AAAAGGCCGC GTTGCTGGCG
       61 TTTTTCCATA GGCTCCGCCC CCCTGACGAG CATCACAAAA ATCGACGCTC AAGTCAGAGG
      121 TGGCGAAACC CGACAGGACT ATAAAGATAC CAGGCGTTTC CCCCTGGAAG CTCCCTCGTG
      181 CGCTCTCCTG TTCCGACCCT GCCGCTTACC GGATACCTGT CCGCCTTTCT CCCTTCGGGA
      241 AGCGTGGCGC TTTCTCATAG CTCACGCTGT AGGTATCTCA GTTCGGTGTA GGTCGTTCGC
      301 TCCAAGCTGG GCTGTGTGCA CGAACCCCCC GTTCAGCCCG ACCGCTGCGC CTTATCCGGT
      361 AACTATCGTC TTGAGTCCAA CCCGGTAAGA CACGACTTAT CGCCACTGGC AGCAGCCACT
      421 GGTAACAGGA TTAGCAGAGC GAGGTATGTA GGCGGTGCTA CAGAGTTCTT GAAGTGGTGG
      481 CCTAACTACG GCTACACTAG AAGAACAGTA TTTGGTATCT GCGCTCTGCT GAAGCCAGTT
      541 ACCTTCGGAA AAAGAGTTGG TAGCTCTTGA TCCGGCAAAC AAACCACCGC TGGTAGCGGT
      601 GGTTTTTTTG TTTGCAAGCA GCAGATTACG CGCAGAAAAA AAGGATCTCA AGAAGATCCT
```

661 TTGATCTTTT CTACGGGGTC TGACGCTCAG TGGAACGACG CGTAACTCAC GTTAAGGGAT
721 TTTGGTCATG AGCTTGCGCC GTCCCGTCAA GTCAGCGTAA TGCTCTGCCA GTGTTACAAC
781 CAATTAACCA ATTCTGATTA GAAAAACTCA TCGAGCATCA AATGAAACTG CAATTTATTC
841 ATATCAGGAT TATCAATACC ATATTTTTGA AAAAGCCGTT TCTGTAATGA AGGAGAAAAC
901 TCACCGAGGC AGTTCCATAG GATGGCAAGA TCCTGGTATC GGTCTGCGAT TCCGACTCGT
961 CCAACATCAA TACAACCTAT TAATTTCCCC TCGTCAAAAA TAAGGTTATC AAGTGAGAAA
1021 TCACCATGAG TGACGACTGA ATCCGGTGAG AATGGCAAAA GTTTATGCAT TTCTTTCCAG
1081 ACTTGTTCAA CAGGCCAGCC ATTACGCTCG TCATCAAAAT CACTCGCATC AACCAAACCG
1141 TTATTCATTC GTGATTGCGC CTGAGCGAGA CGAAATACGC GATCGCTGTT AAAAGGACAA
1201 TTACAAACAG GAATCGAATG CAACCGGCGC AGGAACACTG CCAGCGCATC AACAATATTT
1261 TCACCTGAAT CAGGATATTC TTCTAATACC TGGAATGCTG TTTTTCCGGG GATCGCAGTG
1321 GTGAGTAACC ATGCATCATC AGGAGTACGG ATAAAATGCT TGATGGTCGG AAGAGGCATA
1381 AATTCCGTCA GCCAGTTTAG TCTGACCATC TCATCTGTAA CATCATTGGC AACGCTACCT
1441 TTGCCATGTT TCAGAAACAA CTCTGGCGCA TCGGGCTTCC CATACAAGCG ATAGATTGTC
1501 GCACCTGATT GCCCGACATT ATCGCGAGCC CATTTATACC CATATAAATC AGCATCCATG
1561 TTGGAATTTA ATCGCGGCCT CGACGTTTCC CGTTGAATAT GGCTCATAAC ACCCCTTGTA
1621 TTACTGTTTA TGTAAGCAGA CAGTTTTATT GTTCATGATG ATATTATTTT ATCTTGTGCA
1681 ATGTAACATC AGAGATTTTG AGACACGGGC CAGAGCTGCC AGGAAACAGC TATGACCATG
1741 TAATACGACT CACTATAGGG GATATCAGCT GGATGGCAAA TAATGATTTT ATTTTGACTG
1801 ATAGTGACCT GTTCGTTGCA ACAAATTGAT AAGCAATGCT TTCTTATAAT GCCAACTTTG
1861 TACAAGAAAG CTGGGTGATG GCTGGCAACT AGAAGGCACA GTCGAGGCTG ATCAGCGAGC
1921 TCTAGCATTT AGGTGACACT ATAGAATAGG GCCCTCTAGA TGCATGCTCG ACTGGATCCG
1981 GTACCAAGGT CGGGCAGGAA GAGGGCCTAT TTCCCATGAT TCCTTCATAT TTGCATATAC
2041 GATACAAGGC TGTTAGAGAG ATAATTAGAA TTAATTTGAC TGTAAACACA AAGATATTAG
2101 TACAAAATAC GTGACGTAGA AAGTAATAAT TTCTTGGGTA GTTTGCAGTT TTAAAATTAT
2161 GTTTTAAAAT GGACTATCAT ATGCTTACCG TAACTTGAAA GTATTTCGAT TTCTTGGCTT
2221 TATATATCTT GTGGAAAGGA CGAAACACCG GGTCTTCGCA CGACAGGTTT CCCGACTGGA
2281 AAGCGGGCAG TGAGCGCAAC GCAATTAATG TGAGTTAGCT CACTCATTAG GCACCCCAGG
2341 CTTTACACTT TATGCTTCCG GCTCGTATGT TGTGTGGAAT TGTGAGCGGA TAACAATTTC
2401 ACACAGGAAA CAGCTATGAC CATGATTACG GATTCACTGG CCGTCGTTTT ACAACGTCGT
2461 GACTGGGAAA ACCCTGGCGT TACCCAACTT AATCGCCTTG CAGCACATCC CCCTTTCGCC
2521 AGCTGGCGTA ATAGCGAAGA GGCCCGCACC GATCGCCCTT CCCAACAGTT GCGCAGCCTG
2581 AATGGCGAAT GGCGCGAACG ATATCGTGCG GATCCAGAAG ACCTGTTTTA GAGCTAGAAA
2641 TAGCAAGTTA AAATAAGGCT AGTCCGTTAT CAACTTGAAA AAGTGGCACC GAGTCGGTGC
2701 TTTTTTTCTA GACCCAGCTT TCTTGTACAA AGTTGGCATT AAAGGGCGAA TTCTGACATT
2761 GATTATTGAC TAGTTATTAA TAGTAATCAA TTACGGGGTC ATTAGTTCAT AGCCCATATA
2821 TGGAGTTCCG CGTTACATAA CTTACGGTAA ATGGCCCGCC TGGCTGACCG CCCAACGACC
2881 CCCGCCCATT GACGTCAATA ATGACGTATG TTCCCATAGT AACGCCAATA GGGACTTTCC
2941 ATTGACGTCA ATGGGTGGAC TATTTACGGT AAACTGCCCA CTTGGCAGTA CATCAAGTGT
3001 ATCATATGCC AAGTACGCCC CCTATTGACG TCAATGACGG TAAATGGCCC GCCTGGCATT
3061 ATGCCCAGTA CATGACCTTA TGGGACTTTC CTACTTGGCA GTACATCTAC GTATTAGTCA
3121 TCGCTATTAC CATGGTGATG CGGTTTTGGC AGTACATCAA TGGGCGTGGA TAGCGGTTTG
3181 ACTCACGGGG ATTTCCAAGT CTCCACCCCA TTGACGTCAA TGGGAGTTTG TTTTGGCACC
3241 AAAATCAACG GGACTTTCCA AAATGTCGTA ACAACTCCGC CCCATTGACG CAAATGGGCG
3301 GTAGGCGTGT ACGGTGGGAG GTCTATATAA GCAGAGCTCT CTGGCTAACT AGAGAACCCA
3361 CTGCTTACTG GCTTATCGAA ATAGACCCGC TCGTTGCAAC AAATTGATGA GCAATGCTTT
3421 TTTATAATGC CAACTCTGTA CAAAAAAGTT GGCGAGGATC GAACCCTTGC CACCATGGTG
3481 AGCAAGGGCG AGGAGCTGAT CAAGGAGAAC ATGCACACCA AGCTGTACCT GACAGGCACC
3541 GTGAACAACC ACTACTTCGA ATGCACCGCC GAAGGGGAGG GCAAGCCCTA CGAGGGCACC
3601 CAGACCAACA GGATCAAGGT GGTGCGCGGA GGCCCCCTGC CGTTCGCATT CGACATCCTG
3661 GCCCCCTGCT TTATGTACGG GAGCAAGACC TTCATTAACC ATCCACCTGA CATTCCCGAT
3721 TATTTTAAGC AGTCCTTCCC TGAGGGCTTC ACATGGGAGA GAACCACCGT TTACGAAGAT
3781 GGGGGCACAC TCACTGCTAC ACAGGACACC AGCCTCCAGG ACGGCTGCCT GATCTACAAC
3841 GTCCAAGTCA GAGGGGAGAA CTTCCCATCC AACGGCCCTG TGATGCAGAA GAAAACACTC
3901 GGCTGGGAGG CCAGTACCGA GACCCTGTAC CCCGCTGACG GCAGCCTGGA AGGCAGACTG
3961 GACTGGGCCC TGAAGCTCGT GGGCGGGGGC CACCTGCACT GCAGACTGGA AACCACATAC
4021 AGATCCAAGA AACCCGCAAA GAACCTCAAG ATGCCCGGCG TCTATTTTAT TGACAGGAGA
4081 CTGGAAAGAA TCAAGGAGGC CGACAATGAG ACCTACGTCG AGCAGCACGA GGTGGCTGTG
4141 GCCAGATACT GCGACCTCCC TAGCAAACTG GGGCACAAAC TTAATGGCAT GGACGAGCTG
4201 TACAAGTAAT CTAGAGGGCT ATGCTAGAGC TCGCTGATCA GCCTCGACTG TGCCTTCTAG

```
4261 TTGCCAGCCA TCTGTTGTTT GCCCCTCCCC CGTGCCTTCC TTGACCCTGG AAGGTGCCAC
4321 TCCCACTGTC CTTTCCTAAT AAAATGAGGA AATTGCATCG CATTGTCTGA GTAGGTGTCA
4381 TTCTATTCTG GGGGGTGGGG TGGGGCAGGA CAGCAAGGGG GAGGATTGGT AAGACAATAG
4441 CAGGCATGCT GGGGATGCGG TGGGCTCTAT GGCTTCTGAG GCGGAAAGAA CCAGCAAGCT
4501 TGGGTCTCCC TATAGTGAGT CAGCCTGCTT TTTTGTACAA AGTTGGCATT ATAAAAAAGC
4561 ATTGCTCATC AATGTGTTGC AACGAACAGG TCACTATCAG TCAAAATAAA ATCATTATTT
4621 GGGGCCCGAG CTTAAGACTG GCCGTCGTTT TACAACGTCG TGACTGGGAA AACATCCATG
4681 CTAGCGTTAA CGCGAGAGTA GGGAACTGCC AGGCATCAAA TAAAACGAAA GGCTCAGTCG
4741 GAAGACTGGG CCTTTCGTTT TATCTGTTGT TTGTCGGTGA ACGCTCTCCT GAGTAGGACA
4801 AATCCGCCGG GAGCGGATTT GAACGTTGTG AAGCAACGGC CCGGAGGGTG GCGGGCAGGA
4861 CGCCCGCCAT AAACTGCCAG GCATCAAACT AAGCAGAAGG CCATCCTGAC GGATGGCCTT
4921 TTTGCGTTTC TACAAACTCT TCCTGGCTAG CGGTACGCGT ATTAATTGCG TTGCGCTCAC
4981 TGCCCGCTTT CCAGTCGGGA AACCTGTCGT GCCAGCTGCA TTAATGAATC GGCCAACGCG
5041 CGGGGAGAGG CGGTTTGCGT ATTGGGCGCT CTTCCGCTTC CTCGCTCACT GACTCGCTGC
5101 GCTCGGTCGT TCGGCTGCGG CGAGCGGTAT CAGCTCACTC AAAGGCGGTA ATACGGTTAT
5161 CCACAGAATC AGGGGATAAC GCAGGAAAG
//
```
