# Supplementary material for: CRISPR/Cas9 Knock-Out in Primary Neonatal and Adult Cardiomyocytes Reveals Distinct cAMP Dynamics Regulation by Various PDE2A and PDE3A Isoforms
Source: Cells. 2023 Jun 4;12(11):1543. doi: 10.3390/cells12111543 (PMC10253201; doi:10.3390/cells12111543)
Supplement: Supplementary file 1 [file cells-12-01543-s001.zip › Supplementary File S2 pDNR221_cas9.pdf]

```
LOCUS       pDNR221_cas9        4608 bp ds-DNA     circular     13-DEC-2016
FEATURES             Location/Qualifiers

     misc_feature    569..668
                     /note="aatL1"
     gene            6760..7569
                     /note="kanamycin resistance gene"
     rep_origin      7690..8363
                     /note="ColE1 (pUC-type) origin of replication"
     misc_feature    6491..6590
                     /note="attL2"
     terminator      726..857
                     /label=SV40_PA_terminator
     repeat_region   718..852
                     /note=""
     terminator      866..997
                     /label=SV40_PA_terminator
     repeat_region   853..992
                     /note=""
     terminator      1006..1137
                     /label=SV40_PA_terminator
     repeat_region   993..1132
                     /note=""
     promoter        1199..1864
                     /label=CMV_immearly_promoter
     misc_feature    1278..1565
                     /label=CAG_enhancer
     source          1890..1895
                     /organism="hCas9"
                     /mol_type="other DNA"
     CDS             1896..6038
                     /note="cas9 DAPCEL optimised"
                     /translation="MDKKYSIGLDIGTNSVGWAVITDEYKVPSKKFKVLGNTDRHSIK
                     KNLIGALLFDSGETAEATRLKRTARRRYTRRKNRICYLQEIFSNEMAKVDDSFFHRLE
                     ESFLVEEDKKHERHPIFGNIVDEVAYHEKYPTIYHLRKKLVDSTDKADLRLIYLALAH
                     MIKFRGHFLIEGDLNPDNSDVDKLFIQLVQTYNQLFEENPINASGVDAKAILSARLSK
                     SRRLENLIAQLPGEKKNGLFGNLIALSLGLTPNFKSNFDLAEDAKLQLSKDTYDDDLD
                     NLLAQIGDQYADLFLAAKNLSDAILLSDILRVNTEITKAPLSASMIKRYDEHHQDLTL
                     LKALVRQQLPEKYKEIFFDQSKNGYAGYIDGGASQEEFYKFIKPILEKMDGTEELLVK
                     LNREDLLRKQRTFDNGSIPHQIHLGELHAILRRQEDFYPFLKDNREKIEKILTFRIPY
                     YVGPLARGNSRFAWMTRKSEETITPWNFEEVVDKGASAQSFIERMTNFDKNLPNEKVL
                     PKHSLLYEYFTVYNELTKVKYVTEGMRKPAFLSGEQKKAIVDLLFKTNRKVTVKQLKE
                     DYFKKIECFDSVEISGVEDRFNASLGTYHDLLKIIKDKDFLDNEENEDILEDIVLTLT
                     LFEDREMIEERLKTYAHLFDDKVMKQLKRRRYTGWGRLSRKLINGIRDKQSGKTILDF
                     LKSDGFANRNFMQLIHDDSLTFKEDIQKAQVSGQGDSLHEHIANLAGSPAIKKGILQT
                     VKVVDELVKVMGRHKPENIVIEMARENQTTQKGQKNSRERMKRIEEGIKELGSQILKE
                     HPVENTQLQNEKLYLYYLQNGRDMYVDQELDINRLSDYDVDHIVPQSFLKDDSIDNKV
                     LTRSDKNRGKSDNVPSEEVVKKMKNYWRQLLNAKLITQRKFDNLTKAERGGLSELDKA
                     GFIKRQLVETRQITKHVAQILDSRMNTKYDENDKLIREVKVITLKSKLVSDFRKDFQF
                     YKVREINNYHHAHDAYLNAVVGTALIKKYPKLESEFVYGDYKVYDVRKMIAKSEQEIG
                     KATAKYFFYSNIMNFFKTEITLANGEIRKRPLIETNGETGEIVWDKGRDFATVRKVLS
                     MPQVNIVKKTEVQTGGFSKESILPKRNSDKLIARKKDWDPKKYGGFDSPTVAYSVLVV
                     AKVEKGKSKKLKSVKELLGITIMERSSFEKNPIDFLEAKGYKEVKKDLIIKLPKYSLF
                     ELENGRKRMLASAGELQKGNELALPSKYVNFLYLASHYEKLKGSPEDNEQKQLFVEQH
                     KHYLDEIIEQISEFSKRVILADANLDKVLSAYNKHRDKPIREQAENIIHLFTLTNLGA
                     PAAFKYFDTTIDRKRYTSTKEVLDATLIHQSITGLYETRIDLSQLGGDSRADPKKKRK
                     V."
     misc_feature    6012..6032
                     /note="SV40 NLS"
     misc_feature    6018..6032
```

```
                     /note="NLS"
     terminator      6077..6347
                     /label=TK_PA_terminator
ORIGIN
        1 CTTTCCTGCG TTATCCCCTG ATTCTGTGGA TAACCGTATT ACCGCCTTTG AGTGAGCTGA
       61 TACCGCTCGC CGCAGCCGAA CGACCGAGCG CAGCGAGTCA GTGAGCGAGG AAGCGGAAGA
      121 GCGCCCAATA CGCAAACCGC CTCTCCCCGC GCGTTGGCCG ATTCATTAAT GCAGCTGGCA
      181 CGACAGGTTT CCCGACTGGA AAGCGGGCAG TGAGCGCAAC GCAATTAATA CGCGTACCGC
      241 TAGCCAGGAA GAGTTTGTAG AAACGCAAAA AGGCCATCCG TCAGGATGGC CTTCTGCTTA
      301 GTTTGATGCC TGGCAGTTTA TGGCGGGCGT CCTGCCCGCC ACCCTCCGGG CCGTTGCTTC
      361 ACAACGTTCA AATCCGCTCC CGGCGGATTT GTCCTACTCA GGAGAGCGTT CACCGACAAA
      421 CAACAGATAA AACGAAAGGC CCAGTCTTCC GACTGAGCCT TTCGTTTTAT TTGATGCCTG
      481 GCAGTTCCCT ACTCTCGCGT TAACGCTAGC ATGGATGTTT TCCCAGTCAC GACGTTGTAA
      541 AACGACGGCC AGTCTTAAGC TCGGGCCCCA AATAATGATT TTATTTTGAC TGATAGTGAC
      601 CTGTTCGTTG CAACACATTG ATGAGCAATG CTTTTTTATA ATGCCAACTT TGTACAAAAA
      661 AGCAGGCTGA CTCACTATAG GGAGACCCAA GCTTAATGTA TGCTATACGA AGTTATTTTG
      721 TTAACTTGTT TATTGCAGCT TATAATGGTT ACAAATAAAG CAATAGCATC ACAAATTTCA
      781 CAAATAAAGC ATTTTTTTCA CTGCATTCTA GTTGTGGTTT GTCCAAACTC ATCAATGTAT
      841 CTTATCATGT CTGGATCTTG TTAACTTGTT TATTGCAGCT TATAATGGTT ACAAATAAAG
      901 CAATAGCATC ACAAATTTCA CAAATAAAGC ATTTTTTTCA CTGCATTCTA GTTGTGGTTT
      961 GTCCAAACTC ATCAATGTAT CTTATCATGT CTGGATCTTG TTAACTTGTT TATTGCAGCT
     1021 TATAATGGTT ACAAATAAAG CAATAGCATC ACAAATTTCA CAAATAAAGC ATTTTTTTCA
     1081 CTGCATTCTA GTTGTGGTTT GTCCAAACTC ATCAATGTAT CTTATCATGT CTGGATCTGC
     1141 AGATATAACT TCGTATAATG TATGCTATAC GAAGTTATTA ACTCGAGGTC ACGCGTTGAC
     1201 ATTGATTATT GACTAGTTAT TAATAGTAAT CAATTACGGG GTCATTAGTT CATAGCCCAT
     1261 ATATGGAGTT CCGCGTTACA TAACTTACGG TAAATGGCCC GCCTGGCTGA CCGCCCAACG
     1321 ACCCCCGCCC ATTGACGTCA ATAATGACGT ATGTTCCCAT AGTAACGCCA ATAGGGACTT
     1381 TCCATTGACG TCAATGGGTG GAGTATTTAC GGTAAACTGC CCACTTGGCA GTACATCAAG
     1441 TGTATCATAT GCCAAGTACG CCCCCTATTG ACGTCAATGA CGGTAAATGG CCCGCCTGGC
     1501 ATTATGCCCA GTACATGACC TTATGGGACT TTCCTACTTG GCAGTACATC TACGTATTAG
     1561 TCATCGCTAT TACCATGGTG ATGCGGTTTT GGCAGTACAT CAATGGGCGT GGATAGCGGT
     1621 TTGACTCACG GGGATTTCCA AGTCTCCACC CCATTGACGT CAATGGGAGT TTGTTTTGGC
     1681 ACCAAAATCA ACGGGACTTT CCAAAATGTC GTAACAACTC CGCCCCATTG ACGCAAATGG
     1741 GCGGTAGGCG TGTACGGTGG GAGGTCTATA TAAGCAGAGC TCGTTTAGTG AACCGTCAGA
     1801 TCGCCTGGAG ACGCCATCCA CGCTGTTTTG ACCTCCATAG AAGACACCGG GACCGATCCA
     1861 GCCTCCGGAC TCTAGAGGAT CGAACCCTTG CCACCATGGA CAAAAAGTAT TCTATAGGTC
     1921 TGGACATCGG TACAAACTCG GTTGGATGGG CGGTCATCAC CGACGAGTAC AAAGTGCCGT
     1981 CCAAGAAGTT CAAGGTGTTA GGCAACACCG ACCGGCATAG CATCAAGAAG AACCTCATCG
     2041 GGGCCCTCCT GTTCGACAGC GGCGAGACCG CGGAGGCGAC CAGGCTAAAG CGCACCGCCA
     2101 GGCGGCGCTA CACCAGGCGC AAGAACAGGA TCTGCTACTT GCAAGAGATC TTCTCCAACG
     2161 AGATGGCGAA GGTCGACGAC AGCTTCTTCC ACCGCCTCGA GGAGTCCTTC CTCGTCGAGG
     2221 AGGACAAGAA GCACGAGAGG CACCCCATCT TCGGCAACAT CGTCGACGAG GTGGCCTACC
     2281 ACGAGAAGTA CCCCACCATC TACCACTTAC GCAAGAAGCT CGTCGACTCC ACCGACAAGG
     2341 CGGACCTCCG GCTGATCTAC CTCGCACTGG CGCACATGAT CAAGTTCAGG GGCCACTTCC
     2401 TCATCGAGGG CGACCTGAAC CCCGACAACA GCGACGTCGA CAAGTTGTTC ATCCAACTCG
     2461 TCCAGACTTA TAACCAGCTG TTCGAGGAGA ACCCCATCAA CGCCAGCGGC GTCGACGCCA
     2521 AGGCGATCCT CTCCGCCCGC CTCAGCAAGT CCCGGCGCCT GGAGAACCTA ATCGCCCAAC
     2581 TACCGGGCGA GAAGAAGAAC GGGCTGTTCG GGAACCTAAT CGCCCTCTCC CTCGGCCTCA
     2641 CTCCCAACTT CAAGTCCAAC TTCGACCTCG CCGAGGACGC CAAGCTGCAA CTCTCCAAGG
     2701 ACACCTATGA CGACGACTTA GACAACCTGC TCGCGCAGAT CGGCGACCAG TACGCCGACC
     2761 TCTTCCTCGC CGCCAAGAAC CTGTCCGACG CCATCCTGCT CTCCGACATC TTGCGGGTCA
     2821 ACACCGAGAT CACCAAGGCC CCGTTGTCCG CCTCCATGAT CAAGCGGTAT GACGAGCACC
     2881 ACCAGGACCT CACCCTCCTG AAGGCCCTGG TCCGCCAGCA GCTCCCCGAG AAGTACAAGG
     2941 AGATCTTCTT CGACCAGTCC AAGAACGGCT ACGCCGGCTA CATCGACGGG GGCGCCTCGC
     3001 AGGAGGAGTT CTACAAGTTC ATCAAGCCCA TCTTAGAGAA GATGGACGGC ACCGAGGAGC
     3061 TGCTCGTCAA GTTGAACAGG GAGGACCTCT TACGGAAGCA GCGCACTTTC GACAACGGGT
     3121 CCATCCCGCA CCAGATCCAT CTCGGCGAGT TACACGCCAT CCTCCGGCGG CAGGAGGACT
     3181 TCTACCCCTT CCTGAAGGAC AACAGGGAGA AGATCGAGAA GATCCTCACC TTCCGCATCC
     3241 CCTACTACGT GGGCCCCCTC GCGAGGGGGA ACAGCAGGTT CGCCTGGATG ACCCGCAAGT
     3301 CCGAGGAGAC CATCACTCCC TGGAACTTCG AGGAGGTGGT CGACAAGGGC GCCTCCGCCC
```

3361 AGTCCTTCAT CGAGCGGATG ACCAACTTCG ACAAGAACCT CCCCAACGAG AAGGTCTTGC
3421 CCAAGCACAG CCTCCTCTAC GAATACTTCA CGGTGTACAA CGAGCTCACC AAGGTCAAGT
3481 ACGTGACCGA GGGCATGCGC AAGCCCGCCT TCCTCTCCGG CGAGCAAAAG AAGGCCATCG
3541 TGGACCTGCT ATTCAAGACC AACCGCAAGG TCACTGTGAA GCAGCTGAAG GAGGACTACT
3601 TCAAGAAGAT CGAGTGCTTC GACAGCGTGG AGATCTCCGG CGTGGAGGAC AGATTCAACG
3661 CCTCCCTGGG CACTTATCAC GACCTCTTGA AGATCATCAA GGACAAGGAC TTCCTCGACA
3721 ACGAGGAGAA CGAGGACATC CTGGAGGACA TCGTGCTGAC CCTCACTCTG TTCGAGGACC
3781 GCGAGATGAT CGAGGAGCGG CTCAAGACCT ACGCCCATCT ATTCGACGAC AAGGTCATGA
3841 AGCAACTCAA GAGGCGGAGG TACACTGGCT GGGGCAGGCT CTCCCGCAAG CTCATCAACG
3901 GGATCCGCGA CAAGCAGTCC GGGAAGACCA TCCTGGACTT CCTCAAGTCC GACGGCTTCG
3961 CCAACCGGAA CTTCATGCAA TTAATCCACG ACGACAGCCT CACCTTCAAG GAGGACATCC
4021 AAAAGGCCCA GGTCTCCGGC CAGGGGGACA GCCTGCACGA GCACATCGCC AACCTGGCCG
4081 GCTCGCCCGC CATCAAGAAG GGCATCCTGC AAACCGTCAA GGTGGTGGAC GAGCTCGTCA
4141 AGGTCATGGG GCGCCACAAG CCCGAGAACA TCGTGATCGA GATGGCCAGG GAGAACCAAA
4201 CCACCCAGAA GGGGCAAAAG AACTCGCGCG AGAGGATGAA GCGCATCGAG GAGGGCATCA
4261 AGGAGCTGGG CAGCCAAATC CTCAAGGAGC ACCCCGTGGA GAACACCCAG CTCCAGAACG
4321 AGAAGCTATA CCTATACTAC CTACAGAACG GCCGGGACAT GTACGTCGAC CAGGAGTTAG
4381 ACATCAACAG GCTGAGCGAC TACGACGTCG ACCATATCGT GCCCCAGAGC TTCCTCAAGG
4441 ACGACTCCAT AGATAACAAG GTCCTGACGA GGTCCGACAA GAACAGGGGC AAGTCGGACA
4501 ACGTGCCCAG CGAGGAGGTC GTCAAGAAGA TGAAGAACTA CTGGCGGCAG CTCTTGAACG
4561 CCAAGCTGAT CACCCAGAGG AAGTTCGACA ACCTGACGAA GGCCGAGAGG GGCGGCCTCA
4621 GCGAGCTCGA CAAGGCCGGC TTCATCAAGC GGCAGCTCGT GGAGACCCGG CAGATCACCA
4681 AGCACGTCGC CCAGATCCTC GACAGCCGGA TGAACACCAA GTATGACGAG AACGACAAGC
4741 TCATCCGCGA GGTGAAGGTC ATCACTCTGA AGTCCAAGCT GGTGTCCGAC TTCCGCAAGG
4801 ACTTCCAGTT CTACAAGGTC AGGGAGATCA ACAACTATCA CCACGCACAC GATGCGTACT
4861 TGAACGCCGT CGTGGGCACC GCCCTCATCA AGAAGTACCC CAAGCTCGAG TCGGAGTTCG
4921 TCTACGGCGA CTACAAGGTG TACGACGTGA GGAAGATGAT CGCCAAGTCC GAGCAGGAGA
4981 TCGGGAAGGC CACTGCCAAG TACTTCTTCT ATTCCAACAT CATGAACTTC TTCAAGACCG
5041 AGATCACCCT CGCCAACGGC GAGATCCGGA AGCGGCCCTT GATCGAGACC AACGGGGAGA
5101 CCGGCGAGAT CGTCTGGGAC AAGGGGCGCG ACTTCGCCAC CGTCCGGAAG GTCCTCTCGA
5161 TGCCGCAGGT CAACATCGTC AAGAAGACCG AGGTCCAAAC CGGGGGCTTC TCGAAGGAGT
5221 CCATCCTGCC CAAGCGGAAC TCGGACAAGC TCATCGCCAG GAAGAAGGAC TGGGACCCCA
5281 AGAAGTACGG CGGCTTCGAC AGCCCCACGG TCGCCTACTC CGTCTTGGTC GTGGCCAAGG
5341 TCGAGAAGGG GAAGTCGAAG AAGCTGAAGT CGGTGAAGGA GCTGTTGGGG ATCACCATCA
5401 TGGAGCGGAG CTCGTTCGAG AAGAACCCGA TCGACTTCCT GGAGGCCAAG GGCTACAAGG
5461 AGGTGAAGAA GGACCTGATC ATCAAGTTGC CCAAGTACAG CCTCTTCGAG CTGGAGAACG
5521 GCAGGAAGCG CATGTTAGCC AGCGCCGGCG AGCTGCAGAA GGGCAACGAG TTAGCCTTAC
5581 CCTCGAAGTA CGTCAACTTC CTGTACCTGG CCAGCCACTA CGAGAAGCTC AAGGGCAGCC
5641 CCGAGGACAA CGAGCAAAAG CAACTCTTCG TCGAGCAACA CAAGCACTAC CTGGACGAGA
5701 TCATCGAGCA GATCAGCGAG TTCTCCAAGA GGGTGATCCT GGCCGACGCC AACCTGGACA
5761 AGGTGCTCAG CGCCTACAAC AAGCACCGGG ACAAGCCCAT CAGGGAGCAG GCCGAGAACA
5821 TCATCCACCT GTTCACGCTC ACGAACCTCG GCGCCCCGGC CGCCTTCAAG TACTTCGACA
5881 CCACCATCGA CAGGAAGCGC TACACGTCCA CCAAGGAGGT GCTGGACGCC ACCCTCATCC
5941 ACCAGTCGAT CACCGGCCTC TACGAGACCC GGATCGACCT CAGCCAATTG GGCGGCGACA
6001 GCAGGGCTGA CCCCAAGAAA AAGAGGAAAG TCTGATAAAA GGGTTCGATC CCTACCGGTT
6061 AGTAATGAGT TTAAACGGGG GAGGCTAACT GAAACACGGA AGGAGACAAT ACCGGAAGGA
6121 ACCCGCGCTA TGACGGCAAT AAAAAGACAG AATAAAACGC ACGGGTGTTG GGTCGTTTGT
6181 TCATAAACGC GGGGTTCGGT CCCAGGGCTG GCACTCTGTC GATACCCCAC CGAGACCCCA
6241 TTGGGGCCAA TACGCCCGCG TTTCTTCCTT TTCCCCACCC CACCCCCCAA GTTCGGGTGA
6301 AGGCCCAGGG CTCGCAGCCA ACGTCGGGGC GGCAGGCCCT GCCATAGCAG ATCTGCGCAG
6361 CTGGGGCTCT AGGGGGTATC CCCACGCGCC CTGTCTCGAG CATGCATCTA GAGGGCCCTA
6421 TTCTATAGTG TCACCTAAAT GCTAGAGCTC GCTGATCAGC CTCGACTGTG CCTTCTAGTT
6481 GCCAGCCATC ACCCAGCTTT CTTGTACAAA GTTGGCATTA TAAGAAAGCA TTGCTTATCA
6541 ATTTGTTGCA ACGAACAGGT CACTATCAGT CAAAATAAAA TCATTATTTG CCATCCAGCT
6601 GATATCCCCT ATAGTGAGTC GTATTACATG GTCATAGCTG TTTCCTGGCA GCTCTGGCCC
6661 GTGTCTCAAA ATCTCTGATG TTACATTGCA CAAGATAAAA TAATATCATC ATGAACAATA
6721 AAACTGTCTG CTTACATAAA CAGTAATACA AGGGGTGTTA TGAGCCATAT TCAACGGGAA
6781 ACGTCGAGGC CGCGATTAAA TTCCAACATG GATGCTGATT TATATGGGTA TAAATGGGCT
6841 CGCGATAATG TCGGGCAATC AGGTGCGACA ATCTATCGCT TGTATGGGAA GCCCGATGCG
6901 CCAGAGTTGT TTCTGAAACA TGGCAAAGGT AGCGTTGCCA ATGATGTTAC AGATGAGATG

6961 GTCAGACTAA ACTGGCTGAC GGAATTTATG CCTCTTCCGA CCATCAAGCA TTTTATCCGT
7021 ACTCCTGATG ATGCATGGTT ACTCACCACT GCGATCCCCG GAAAAACAGC ATTCCAGGTA
7081 TTAGAAGAAT ATCCTGATTC AGGTGAAAAT ATTGTTGATG CGCTGGCAGT GTTCCTGCGC
7141 CGGTTGCATT CGATTCCTGT TTGTAATTGT CCTTTTAACA GCGATCGCGT ATTTCGTCTC
7201 GCTCAGGCGC AATCACGAAT GAATAACGGT TTGGTTGATG CGAGTGATTT TGATGACGAG
7261 CGTAATGGCT GGCCTGTTGA ACAAGTCTGG AAAGAAATGC ATAAACTTTT GCCATTCTCA
7321 CCGGATTCAG TCGTCACTCA TGGTGATTTC TCACTTGATA ACCTTATTTT TGACGAGGGG
7381 AAATTAATAG GTTGTATTGA TGTTGGACGA GTCGGAATCG CAGACCGATA CCAGGATCTT
7441 GCCATCCTAT GGAACTGCCT CGGTGAGTTT TCTCCTTCAT TACAGAAACG GCTTTTTCAA
7501 AAATATGGTA TTGATAATCC TGATATGAAT AAATTGCAGT TTCATTTGAT GCTCGATGAG
7561 TTTTTCTAAT CAGAATTGGT TAATTGGTTG TAACACTGGC AGAGCATTAC GCTGACTTGA
7621 CGGGACGGCG CAAGCTCATG ACCAAAATCC CTTAACGTGA GTTACGCGTC GTTCCACTGA
7681 GCGTCAGACC CCGTAGAAAA GATCAAAGGA TCTTCTTGAG ATCCTTTTTT TCTGCGCGTA
7741 ATCTGCTGCT TGCAAACAAA AAAACCACCG CTACCAGCGG TGGTTTGTTT GCCGGATCAA
7801 GAGCTACCAA CTCTTTTTCC GAAGGTAACT GGCTTCAGCA GAGCGCAGAT ACCAAATACT
7861 GTTCTTCTAG TGTAGCCGTA GTTAGGCCAC CACTTCAAGA ACTCTGTAGC ACCGCCTACA
7921 TACCTCGCTC TGCTAATCCT GTTACCAGTG GCTGCTGCCA GTGGCGATAA GTCGTGTCTT
7981 ACCGGGTTGG ACTCAAGACG ATAGTTACCG GATAAGGCGC AGCGGTCGGG CTGAACGGGG
8041 GGTTCGTGCA CACAGCCCAG CTTGGAGCGA ACGACCTACA CCGAACTGAG ATACCTACAG
8101 CGTGAGCTAT GAGAAAGCGC CACGCTTCCC GAAGGGAGAA AGGCGGACAG GTATCCGGTA
8161 AGCGGCAGGG TCGGAACAGG AGAGCGCACG AGGGAGCTTC CAGGGGGAAA CGCCTGGTAT
8221 CTTTATAGTC CTGTCGGGTT TCGCCACCTC TGACTTGAGC GTCGATTTTT GTGATGCTCG
8281 TCAGGGGGGC GGAGCCTATG GAAAAACGCC AGCAACGCGG CCTTTTTACG GTTCCTGGCC
8341 TTTTGCTGGC CTTTTGCTCA CATGTT
//
